# Assessment of bidirectional relationships between hypothyroidism and endometrial cancer: a two-sample Mendelian randomization study

**DOI:** 10.3389/fendo.2024.1308208

**Published:** 2024-05-16

**Authors:** Bolin Wang, Yuxi Luo, Tianxin Liu, Shengnan Xu, Jinli Pei, Jie Liu, Jinming Yu

**Affiliations:** ^1^ Lung Cancer Center, West China Hospital, Sichuan University, Chengdu, Sichuan, China; ^2^ Department of Radiation Oncology and Shandong Provincial Key Laboratory of Radiation Oncology, Shandong Cancer Hospital and Institute, Shandong First Medical University and Shandong Academy of Medical Sciences, Jinan, Shandong, China; ^3^ Research Unit of Radiation Oncology, Chinese Academy of Medical Sciences, Jinan, Shandong, China; ^4^ Department of Radio-immunology and Molecular Imaging Laboratory, Shandong Cancer Hospital and Institute Shandong First Medical University and Shandong Academy of Medical Sciences, Jinan, China; ^5^ College of Clinical Medicine, Southwest Medical University, Luzhou, China

**Keywords:** hypothyroidism, autoimmune hypothyroidism, endometrial cancer, Mendelian randomization, causality

## Abstract

**Objective:**

Hypothyroidism, characterized by reduced thyroid hormone levels, and endometrial cancer, a prevalent gynecological malignancy, have been suggested to have a potential association in previous observational studies. However, the causal relationship between them remains uncertain. This study aimed to investigate the causal relationship between hypothyroidism and endometrial cancer using a bilateral Mendelian randomization approach.

**Methods:**

A bidirectional two-sample Mendelian randomization study was conducted using summary statistics from genome-wide association studies to identify genetic variants associated with hypothyroidism and endometrial cancer. The inverse variance weighting method was used as the main analysis, and sensitivity analyses were conducted to validate the MR results.

**Results:**

The results of our analysis did not support a causal effect of hypothyroidism (OR: 0.93, p=0.08) or autoimmune hypothyroidism (OR: 0.98, p=0.39) on endometrial cancer risk. In the reverse MR analysis, we did not find a significant causal effect of endometrial cancer on hypothyroidism (OR: 0.96, p=0.75) or autoimmune hypothyroidism (OR: 0.92, p=0.50). Based on subgroup analysis by pathological subtypes of endometrial cancer, the above findings were further substantiated (all p-value >0.05).

**Conclusions:**

Our Mendelian randomization analysis suggests a lack of causal association between hypothyroidism and endometrial cancer. To gain a deeper understanding of this association, it is essential to conduct large-scale randomized controlled trials in the future to validate our findings.

## 1 Introduction

Endometrial cancer (EC) is a malignant epithelial tumor originating in the endometrium, ranking as the most common cancer type in developed countries and the second most common in developing countries (1, 2). In 2020, a significant number of newly diagnosed cases were reported worldwide, with 417,367 cases and 97,370 deaths attributed to this disease (2). Notably, China has observed a high incidence of EC, with approximately 84,520 new cases reported in 2022 (3). Unlike other types of cancer that have experienced declining incidence rates over the past two decades, the global incidence of endometrial cancer has continued to rise steadily (1). Furthermore, there is a significant trend towards an earlier age of onset, especially observed in South Africa and specific Asian countries with a considerably high incidence rate (4). Of particular concern is the fact that around 10% to 15% of EC patients are diagnosed at an advanced stage, resulting in a relatively low 5-year survival rate of only 10% to 20% (5). Given these challenges, it is imperative to prioritize the identification of innovative risk factors in order to mitigate the forthcoming healthcare burden.

Hypothyroidism, a prevalent endocrine disorder characterized by reduced thyroid hormone production, affects a significant number of individuals with varying rates of prevalence among women ranging from 0.6% to 12% (6). The relationship between EC and hypothyroidism has been investigated extensively (7). Some studies have found that hypothyroidism is a common comorbidity among patients with endometrial cancer (6, 8). Additionally, it has been observed that 15.3% of EC patients had a prior diagnosis of hypothyroidism, and an additional 8.5% exhibited biochemical evidence of subclinical hypothyroidism based on baseline blood tests (9). Although epidemiological studies suggest a potential link between hypothyroidism and EC, these observations are based on observational data, which are susceptible to confounding factors and bias. Gaining a comprehensive understanding of the causal association between hypothyroidism and endometrial cancer holds significant clinical implications for effective patient management. Regrettably, those previous reports did not provide any information on exact causal relationships between hypothyroidism on endometrial cancer.

Mendelian randomization (MR) is a powerful analytical approach that addresses the limitations inherent in observational methods by employing genetic variants, predominantly single nucleotide polymorphisms (SNPs), as instrumental variables (IVs) to evaluate potential causal associations between exposures and outcomes (10, 11). The random assignment of genetic variants at conception mimics the process of randomization in controlled trials, thereby minimizing the influence of confounding factors (11). Furthermore, as genetic variants precede the onset of disease, they mitigate reverse causation. Importantly, the selected IVs is associated with the exposure but not with any confounders in the exposure-outcome relationship, nor does it exert any other effects on the outcome apart from through the exposure (12). Understanding the causality and biology underlying this association between EC and hypothyroidism is important for deciphering the etiology and can provide therapeutic insights. Therefore, this study aims to contribute to the current knowledge of the causal relationship between endometrial cancer and hypothyroidism using an MR approach to potentially provide a unique perspective.

## 2 Methods

### 2.1 Study design

**Figure 1:**
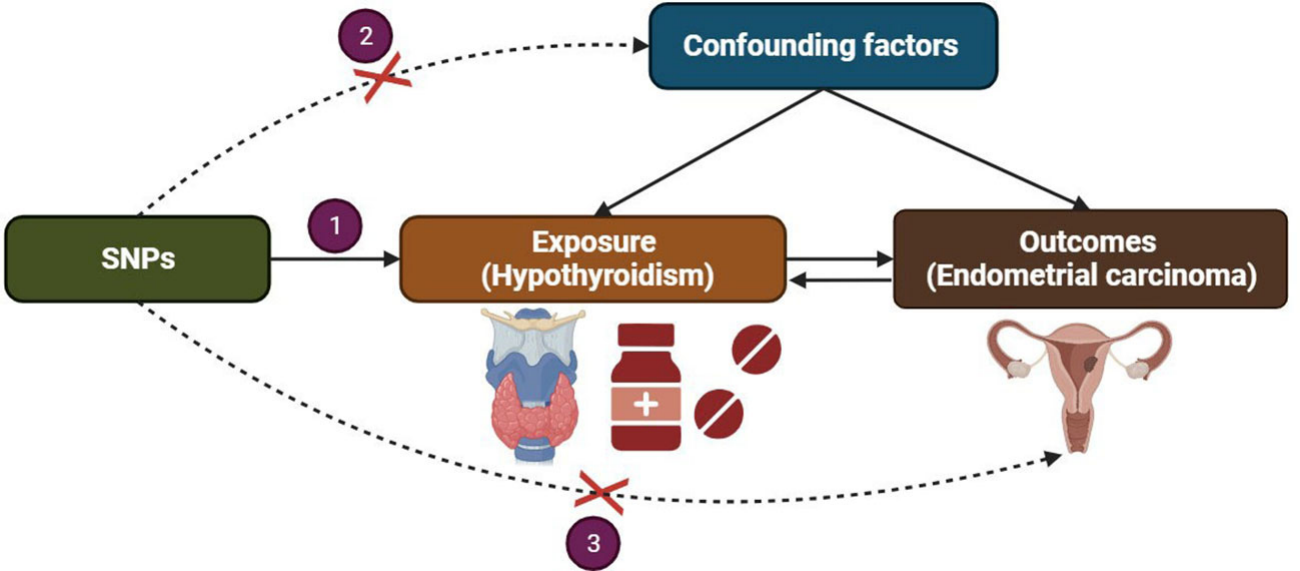
Three assumptions of Mendelian randomization.

We present a concise overview of the bidirectional MR design, as depicted in **Figure 1**. MR analysis is based on three key assumptions (1): The selected instrumental variable, a genetic variant, exhibits a robust association with the exposure; (2) The genetic variant is not associated with any confounding factors; (3) The genetic variants solely influence the outcome through the exposure and not via alternative pathways. To conduct our study, we utilized summary-level data obtained from published genome-wide association studies (GWAS) investigating hypothyroidism and EC (13). Bidirectional MR was performed to evaluate the causal effects of hypothyroidism on EC (forward MR), as well as the effects of EC on hypothyroidism (reverse MR). In the forward MR analysis, we first identified genetic variants associated with hypothyroidism to infer the causal relationship between hypothyroidism and EC. Subsequently, in the reverse MR analysis, we utilized genetic variants associated with EC to infer the causal relationship between EC and hypothyroidism.

### 2.2 Data sources

The summary-level data for the two-sample MR study was obtained from the GWAS database (https://gwas.mrcieu.ac.uk/). Specifically, we utilized data on hypothyroidism (GWAS ID: finn-b-HYPOTHYROIDISM) and autoimmune hypothyroidism (GWAS ID: finn-b-E4_HYTHY_AI_STRICT). Additionally, the data on EC (GWAS ID: ebi-a-GCST006464), endometrioid EC (GWAS ID: ebi-a-GCST006465), and non-endometrioid EC (GWAS ID: ebi-a-GCST006466) was also extracted from the GWAS database. The initial GWAS studies were conducted with approval from the relevant ethics committee, and all participants provided informed consent. Details of the included cohorts were listed in **Supplementary Table S1**.

### 2.3 Selection of genetic instrumental variables

Two thresholds were used to select the IVs. The first threshold selected SNPs less than the genome-wide statistical significance threshold (p < 5 × 10^−8^) to serve as IVs. Unfortunately, after we selected SNPs, only a small number of non-endometrioid EC were selected as IVs, and to explore more relations between hypothyroidism and non-endometrioid EC to obtain more comprehensive results, we used the second threshold that identified SNPs that were smaller than the locus-wide significance level and selected them as the second IVs set to find more potential causal associations (p < 1 × 10^−7^). The independence of SNPs was evaluated using stringent criteria (r^2^ ≤ 0.001; window size =10,000 kb).

### 2.4 Statistical analysis

The primary analysis employed the inverse variance weighted (IVW) method to obtain an unbiased estimate of the causal relationship (12). Additional methods, including the MR Egger and weighted mode, were applied to estimate causal effects under different conditions (14). The weighted median method combined data from multiple genetic variants into a single causal estimate, providing consistent results if at least half of the weight derived from valid instrumental variables (14). The MR-Egger method assessed directional pleiotropy and provided a consistent estimate of the causal effect (15). The intercept of MR-Egger regression was calculated to evaluate horizontal pleiotropy, with a p-value >0.05 indicating weak evidence of pleiotropic effects in the causal analysis (16). Cochran’s Q test, derived from IVW estimation, detected heterogeneity among instrumental variables. Sensitivity analysis was conducted using a leave-one-out method, sequentially removing one SNPs and using the remaining SNPs as instrumental variables for two-sample MR analysis, which assessed the degree of influence of each SNPs on the causal association. Additionally, a reverse-direction MR analysis was performed to investigate the possibility of a reverse-direction causal relationship. The TwoSampleMR package for R software (version 4.2.0) was used for all analyses (17).

## 3 Results

### 3.1 Instrumental variables

All IVs utilized in our analysis exhibited F-statistics exceeding 10, indicating a robust predictive capability for exposure and minimal bias resulting from weak IVs in our investigation. Most of the exposure analyses showed moderate heterogeneity (all p-values for Cochrane’s Q > 0.05), which prompted the adoption of the random-effects IVW MR method. Comprehensive details regarding the chosen SNPs are available in **Supplementary Tables S2**-**S13**.

### 3.2 Causal effect of hypothyroidism on the risk of EC

**Figure 2:**
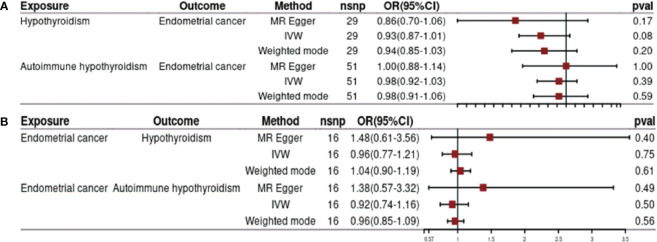
Impact of hypothyroidism on endometrial cancer **(A)** and effects of endometrial cancer on hypothyroidism **(B)**.

Our investigation revealed no significant causal effect of hypothyroidism on the risk of EC [odds ratio (OR) = 0.93, 95% confidence interval (CI): 0.87–1.01, p = 0.08]. Both MR-Egger analysis (OR = 0.86, 95% CI: 0.70–1.06, p = 0.17) and weighted median analysis (OR = 0.94, 95% CI: 0.85–1.03, p = 0.20) supported this finding (**Figure 2A**). Furthermore, we conducted additional analysis to explore the potential causal association between autoimmune hypothyroidism and the risk of EC. Similarly, no causality for genetically predicted autoimmune hypothyroidism on EC risk using the IVW method (OR = 0.98, 95% CI: 0.92–1.03, p = 0.39) (**Figure 2A**). The results obtained from weighted median and MR-Egger analyses were consistent with those from the IVW analysis (all p-values > 0.05) (**Figure 2A**).

### 3.3 Causal effect of EC on the risk of hypothyroidism

**Figure 2B** presents the findings of MR analysis examining the causal effect of EC on the risk of hypothyroidism. We observed genetic liability to EC was not causally associated with risk of hypothyroidism based on IVW analysis (OR = 0.96, 95% CI: 0.77–1.21, p= 0.75). The results of weighted median and MR-Egger are consistent with those of IVW (all p-value > 0.05) (**Figure 2B**). Furthermore, the results from three different MR methods did not reveal any statistically significant associations between EC and autoimmune hypothyroidism examined (all p-values >0.05) (**Figure 2B**).

### 3.4 Subgroup analysis based on pathological subtypes of EC

**Figure 3:**
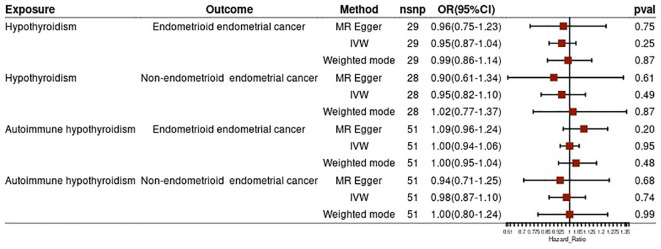
Subgroup analysis of effect of hypothyroidism on endometrial cancer risk.

**Figure 4:**
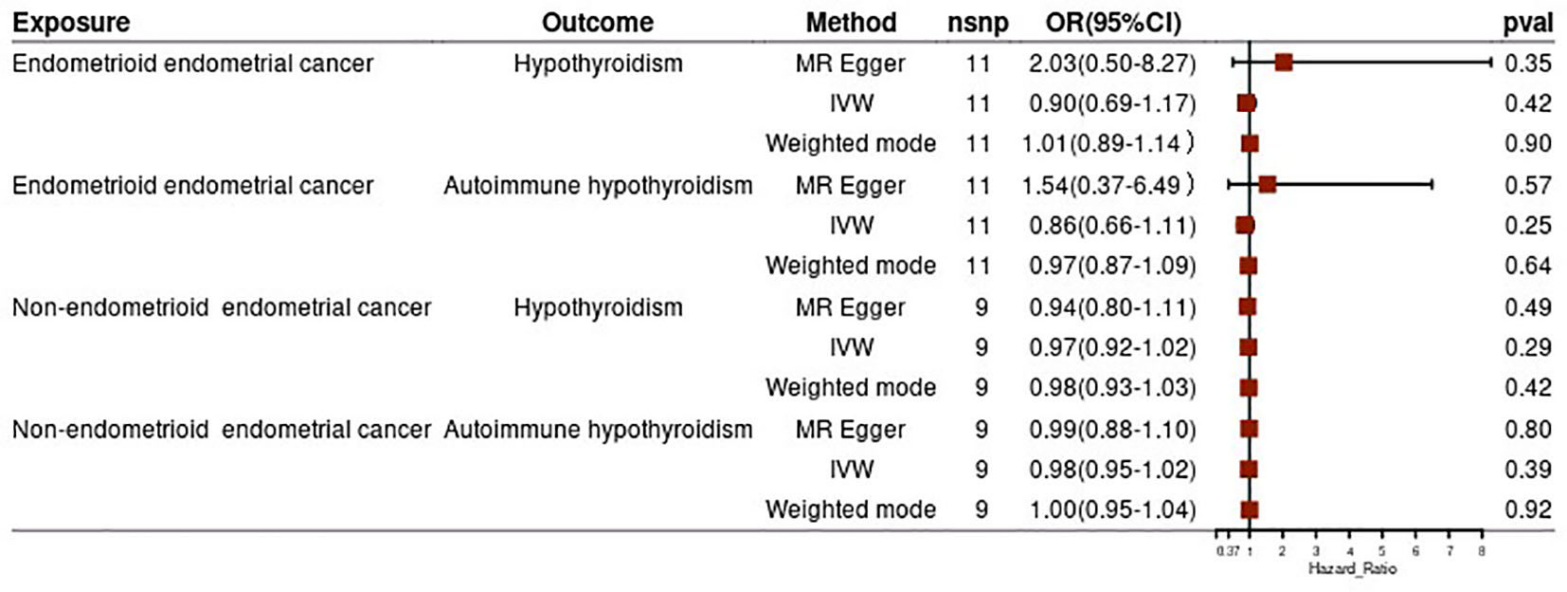
Subgroup analysis of effect of endometrial cancer on hypothyroidism.

To strengthen our findings, we conducted a subgroup analysis based on the pathological subtypes of endometrial cancer. The results from this analysis were consistent with the above findings, suggesting no significant causal effect of hypothyroidism (both general and autoimmune) and different pathological subtypes of endometrial cancer (all p-values >0.05) (**Figure 3**). In the reverse MR analysis, we did not find evidence of a potential causal effect of different pathological subtypes of EC on hypothyroidism or autoimmune hypothyroidism (all p-values >0.05) (**Figure 4**). These subgroup analyses enhance the robustness and reliability of our main findings.

### 3.5 Sensitivity analyses

MR-Egger intercept analysis estimates are presented for sensitivity analyses and indicated the absence of horizontal pleiotropy (all p-values >0.05). These findings affirm the validity and unbiased nature of the instrumental variables employed in our study, unaffected by other genetic or environmental factors. To further validate the aforementioned results, we performed a leave-one-out analysis. The leave-one-out sensitivity analysis demonstrated consistent outcomes across all variables (**Supplementary Figures S1**-**S12**).

## 4 Discussion

Hypothyroidism is a commonly occurring condition characterized by insufficient levels of thyroid hormone (18). Hypothyroidism may contribute to carcinogenesis, as thyroid hormones and TSH possess the capacity to directly stimulate tumorigenesis through various mechanisms encompassing cell surface receptors, estrogen pathways, augmented angiogenesis, and gene expression modification (19). Furthermore, hypothyroidism exhibits associations with diabetes mellitus and cardiovascular disorders, both of which showcase associations with escalated cancer susceptibility (20). Previous studies have investigated the relationship between hypothyroidism and different types of cancer. These studies have found associations between hypothyroidism and decreased risks of breast cancer, thyroid cancer, and hepatocellular carcinoma (6, 21). Unfortunately, they did not report a causal relationship between hypothyroidism and EC risk.

To fill this gap. we conducted first MR analyses to investigate the causal relationship between hypothyroidism and endometrial cancer. Our findings indicate that there is no causal relationship between hypothyroidism or autoimmune hypothyroidism and endometrial cancer. The endometrioid subtype, which accounts for approximately 80% of EC cases, exhibits estrogen responsiveness and typically presents with a favorable prognosis (22, 23). Conversely, the less frequent non-endometrioid subtypes, such as serous and clear cells, are not as responsive to estrogen and are often associated with an unfavorable prognosis (22, 23). To further investigate the potential causal relationships between hypothyroidism and EC susceptibility, we performed subgroup analyses based on above two pathological subtypes. In accordance with the above findings, we also did not find any causal effect of hypothyroidism on the risk of endometrioid or non-endometrioid EC. Furthermore, we also observed any causal effect of endometrioid or non-endometrioid EC on hypothyroidism, which confirmed the robustness of the above findings. In a previous large cohort study involving 1314 EC patients, the relationship between hypothyroidism and the risk of EC was investigated. The results showed that there was no significant association between a history of hypothyroidism and the risk of EC, which in agreement with our finding (24). These findings suggest that factors other than hypothyroidism may play a more prominent role in the pathogenesis of endometrial cancer.

The lack of a causal relationship between hypothyroidism and endometrial cancer observed in our MR analysis contradicts the findings of previous observational studies (7, 9). However, there are several possible reasons for the disparity between our findings and those of previous studies. Firstly, residual confounding may have influenced the results of previous observational studies. These studies rely on observational data, which are susceptible to confounding factors that may distort the association between hypothyroidism and endometrial cancer. By contrast, Mendelian randomization analysis leverages genetic variants as instrumental variables to minimize confounding biases and provide more reliable estimates of causal effects. Secondly, reverse causation is another potential explanation for the discrepancy. Observational studies are vulnerable to reverse causation, as the temporal ordering of events is often challenging to establish definitively. Lastly, measurement errors in the exposure and outcome variables could have contributed to the inconsistent findings. In observational studies, misclassification or inaccuracies in diagnosing hypothyroidism or endometrial cancer can introduce bias into the results, leading to inconsistent associations.

One strength of our study lies in the utilization of the MR method, which offers a valuable framework for evaluating causal relationships and addresses the limitations associated with observational studies. Through the implementation of genetic variants as IVs, we successfully estimated the causal link between EC risk and hypothyroidism while mitigating confounding and reverse causality. To ensure the credibility of our IVs, we exclusively selected SNPs with robust associations and high instrument strength, as indicated by F-statistics exceeding 10. This rigorous criterion bolstered comparability between the exposure and outcome samples, thereby enhancing the reliability of our conclusions. Furthermore, to minimize the impact of sample overlap, we obtained exposure and outcome datasets from different databases, reducing potential interference (25). Additionally, we conducted a comprehensive sensitivity analysis and subgroup analysis. This meticulous examination allowed us to evaluate the robustness and reliability of our findings from various perspectives.

While our study provides robust evidence to refute a direct causal association between hypothyroidism and endometrial cancer, it is essential to acknowledge some limitations. Firstly, our study assumes that the genetic variants used as instrumental variables are independent of potential confounding factors. Although this assumption is reasonable, unmeasured confounders could still impact the estimated results. Additionally, our study focused on European populations, necessitating further research in other ethnic groups to validate our findings. Furthermore, our study did not explore the potential mechanistic pathways that may mediate the observed associations. Future investigations should consider examining how hypothyroidism may indirectly influence endometrial cancer risk through mechanisms such as altered estrogen metabolism or insulin resistance.

While a causal relationship has not been observed between hypothyroidism and endometrial cancer, both may play a role in the progression of each other’s diseases. Dysregulation of immune modulation in the body may potentially explain the intricate relationship between the two. In recent years, Human Leukocyte Antigen-G (HLA-G) has become a research hotspot in studying the relationship between cancer risk and certain autoimmune diseases (26–28). The role of HLA-G has been extensively studied in various inflammatory conditions, especially in autoimmune diseases such as hypothyroidism (28). It is believed that the weakening of the immune suppressive state is one of the factors leading to the development of autoimmune diseases, and HLA-G may serve as a mechanism to counteract the damage in these diseases (28). The expression of HLA-G has been observed to be associated with tumor staging, prognosis, and circulating levels in various types of cancer (29). In endometrial cancer, researchers have also found upregulation of HLA-G expression, and the increased soluble HLA-G levels are associated with advanced pathological staging, metastasis, and poor prognosis in endometrial cancer patients (30, 31). However, there is currently no research definitively stating whether HLA-G is involved in the pathogenesis and progression mechanisms of hypothyroidism and endometrial cancer. Future studies need to further explore this area.

Given the clinical implications of understanding the relationship between hypothyroidism and endometrial cancer, our study contributes valuable insights for individual health management and prevention strategies. While hypothyroidism does not appear to increase the risk of endometrial cancer directly, it is crucial to account for other critical factors when assessing overall risk. Considering the multifactorial nature of endometrial cancer, comprehensive approaches that consider various risk factors such as obesity, diabetes mellitus, postmenopausal estrogen replacement, ovarian dysfunction, infertility, nulliparity, and tamoxifen use are necessary for tailored prevention and early detection strategies (32). Given these limitations, large-scale randomized controlled trials are warranted to validate our findings and provide a deeper understanding of the relationship between hypothyroidism and endometrial cancer. Such trials would help elucidate the causal nature of the association and potentially inform clinical management and interventions for patients with hypothyroidism and endometrial cancer.

## 5 Conclusion

In conclusion, our bidirectional MR study suggests a lack of causal association between hypothyroidism and endometrial cancer. These findings highlight the importance of conducting rigorous randomized controlled trials to elucidate the true nature of this association. Further research is needed to explore other potential risk factors and pathways involved in the development of endometrial cancer, contributing to improved prevention and treatment strategies for this prevalent gynecological malignancy.

## Data availability statement

The original contributions presented in the study are included in the article/**Supplementary Material**. Further inquiries can be directed to the corresponding author.

## Ethics statement

Ethical approval was not required for the study involving humans in accordance with the local legislation and institutional requirements. Written informed consent to participate in this study was not required from the participants or the participants’ legal guardians/next of kin in accordance with the national legislation and the institutional requirements.

## Author contributions

BW: Conceptualization, Data curation, Formal analysis, Investigation, Methodology, Project administration, Resources, Software, Supervision, Validation, Visualization, Writing – original draft, Writing – review & editing. YL: Data curation, Investigation, Methodology, Software, Writing – original draft. TL: Data curation, Formal analysis, Methodology, Software, Writing – original draft. SX: Formal analysis, Methodology, Writing – original draft. JP: Data curation, Formal analysis, Methodology, Writing – original draft. JL: Conceptualization, Formal analysis, Methodology, Resources, Supervision, Validation, Visualization, Writing – original draft, Writing – review & editing. JY: Conceptualization, Investigation, Methodology, Supervision, Validation, Writing – original draft, Writing – review & editing.
