# Supplementary material for: Assessment of bidirectional relationships between hypothyroidism and endometrial cancer: a two-sample Mendelian randomization study
Source: Front Endocrinol (Lausanne). 2024 May 16;15:1308208. doi: 10.3389/fendo.2024.1308208 (PMC11137162; doi:10.3389/fendo.2024.1308208)

**Figure S1** Leave-one-out sensitivity analysis of the impact of hypothyroidism on endometrial cancer risk.

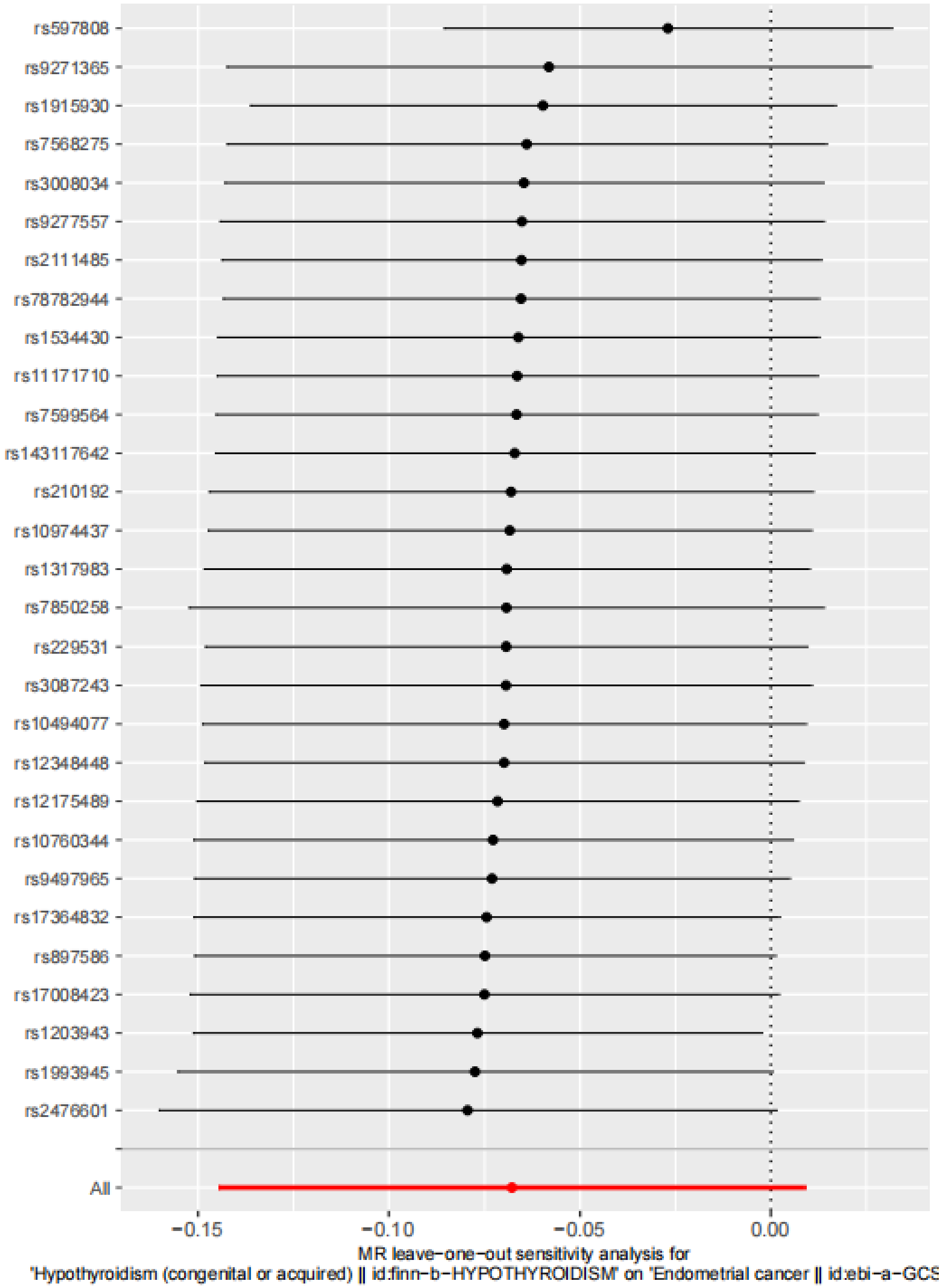

**Figure S2** Leave-one-out sensitivity analysis of the impact of autoimmune hypothyroidism on endometrial cancer risk.

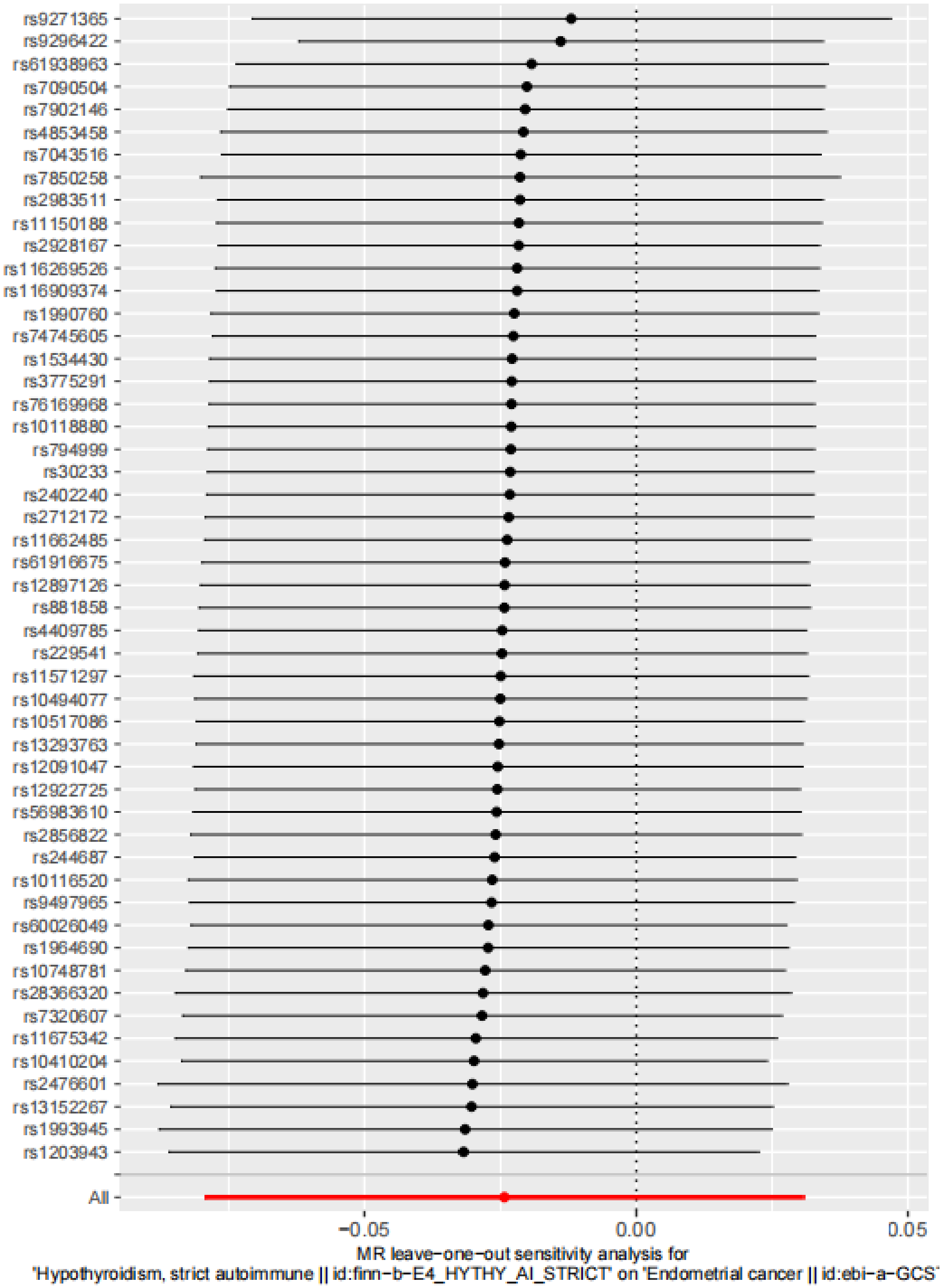

**Figure S3** Leave-one-out sensitivity analysis of the impact of endometrial cancer on hypothyroidism.

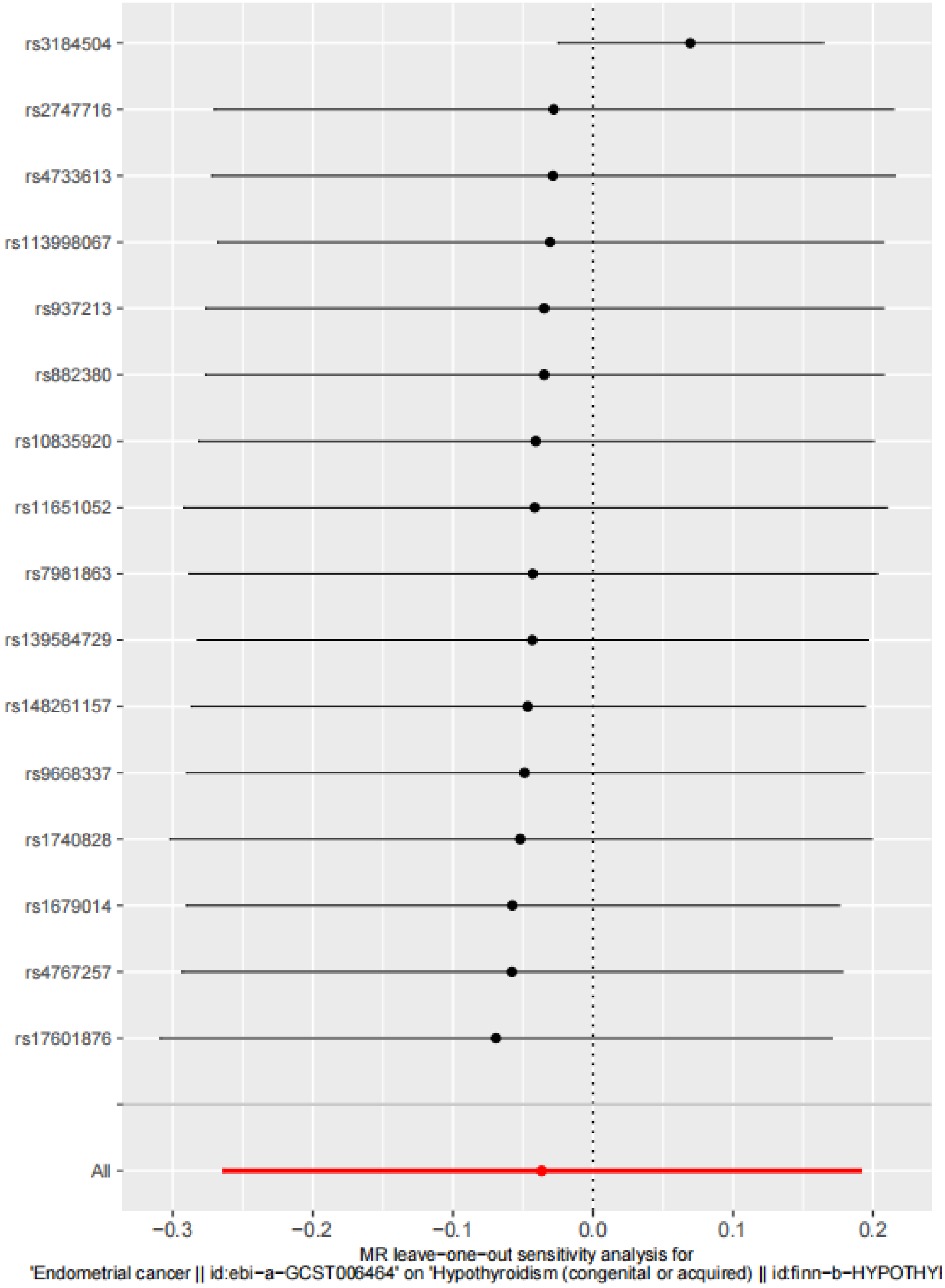

**Figure S4** Leave-one-out sensitivity analysis of the impact of endometrial cancer on autoimmune hypothyroidism.

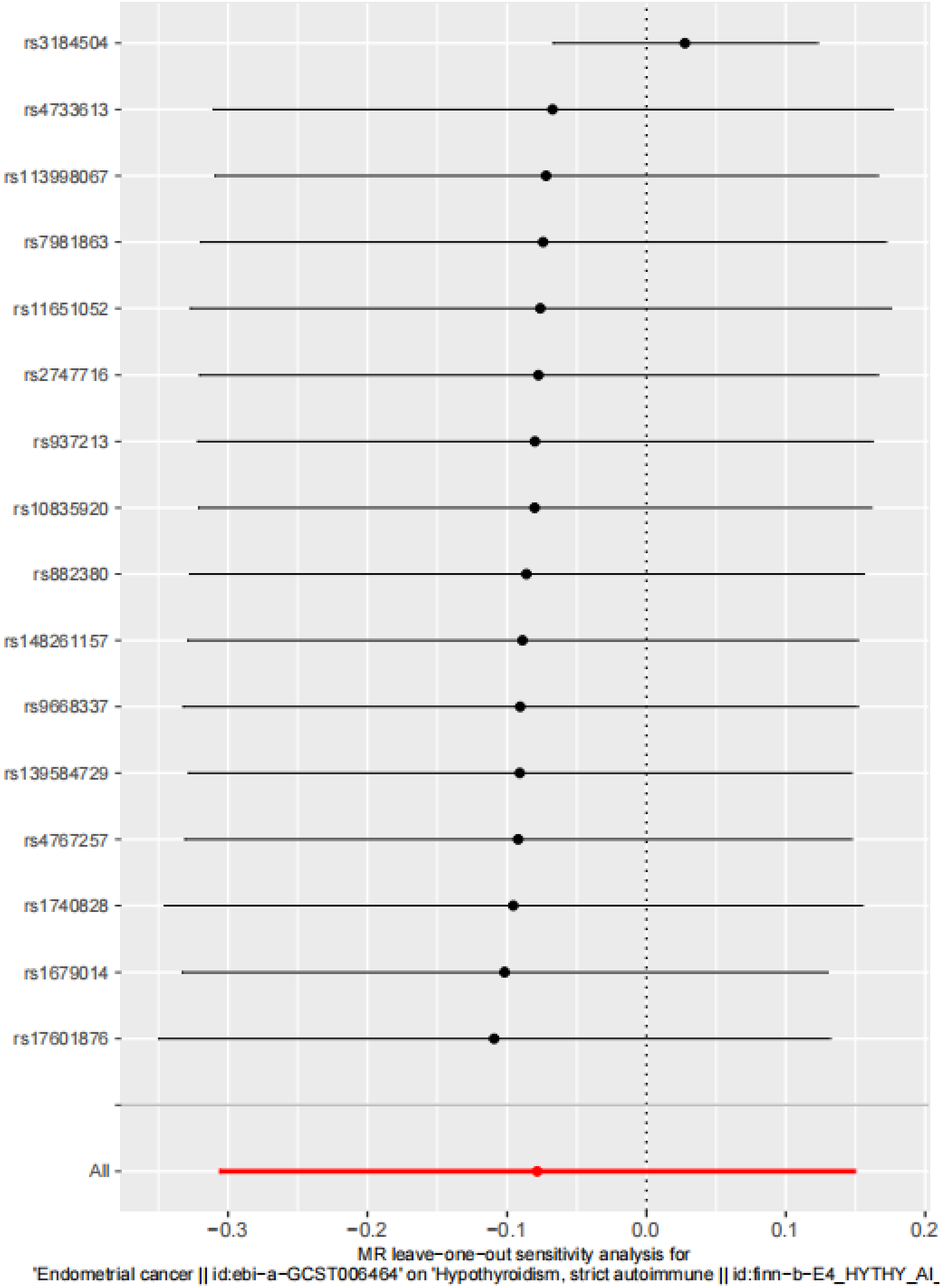

**Figure S5** Leave-one-out sensitivity analysis of the impact of hypothyroidism on endometrioid endometrial cancer risk.

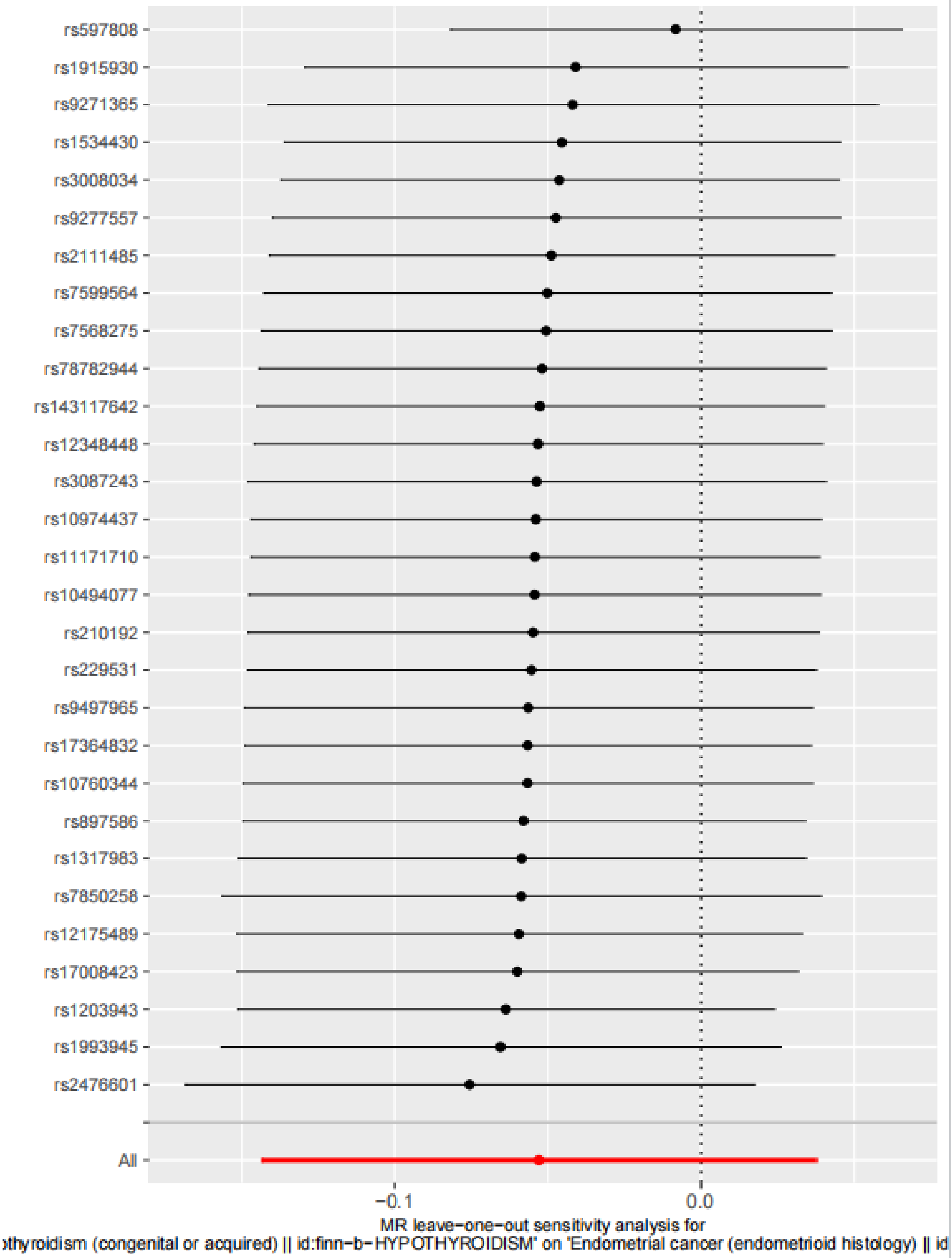

**Figure S6** Leave-one-out sensitivity analysis of the impact of hypothyroidism on non-endometrioid endometrial cancer risk.

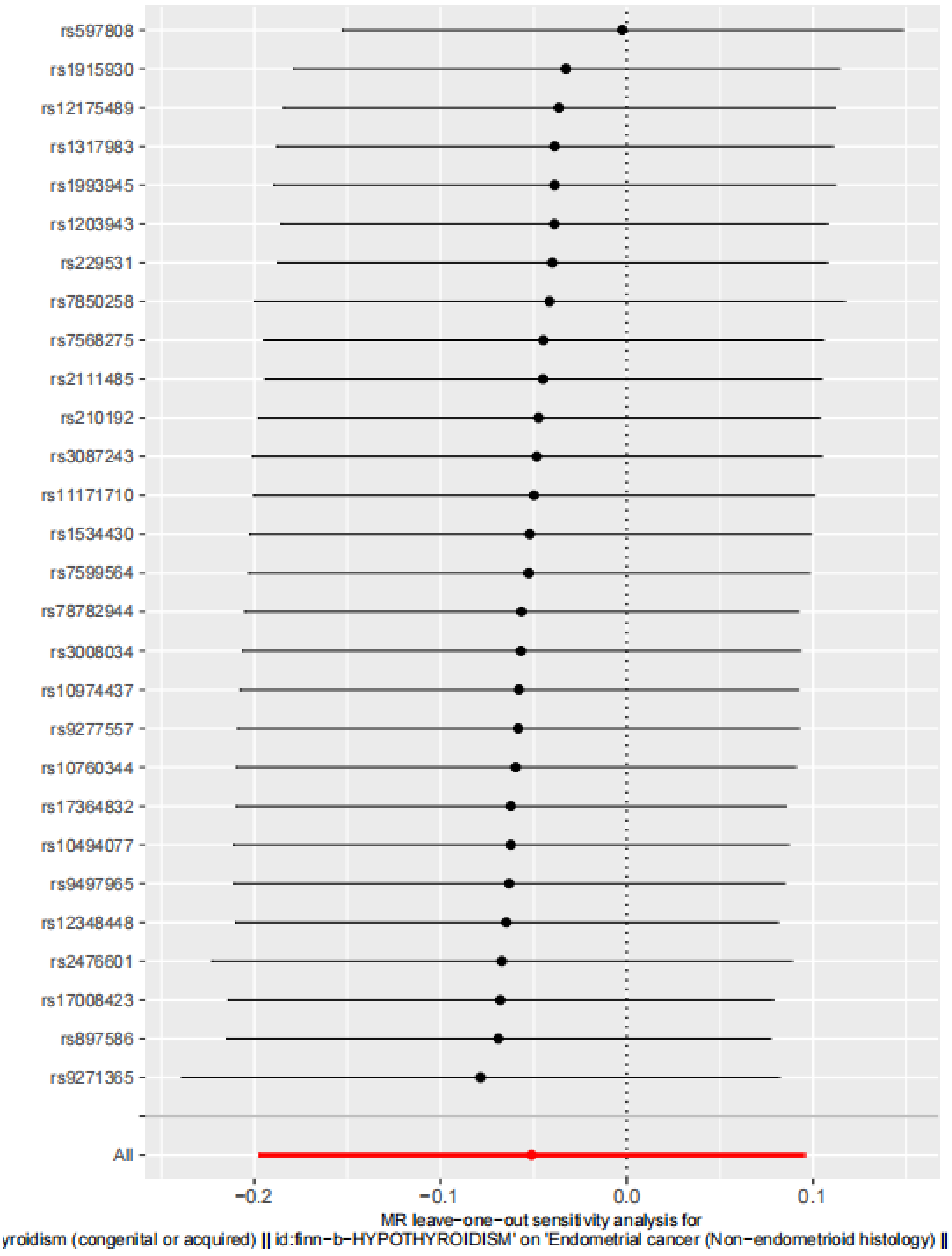

**Figure S7** Leave-one-out sensitivity analysis of the impact of autoimmune hypothyroidism on endometrioid endometrial cancer risk.

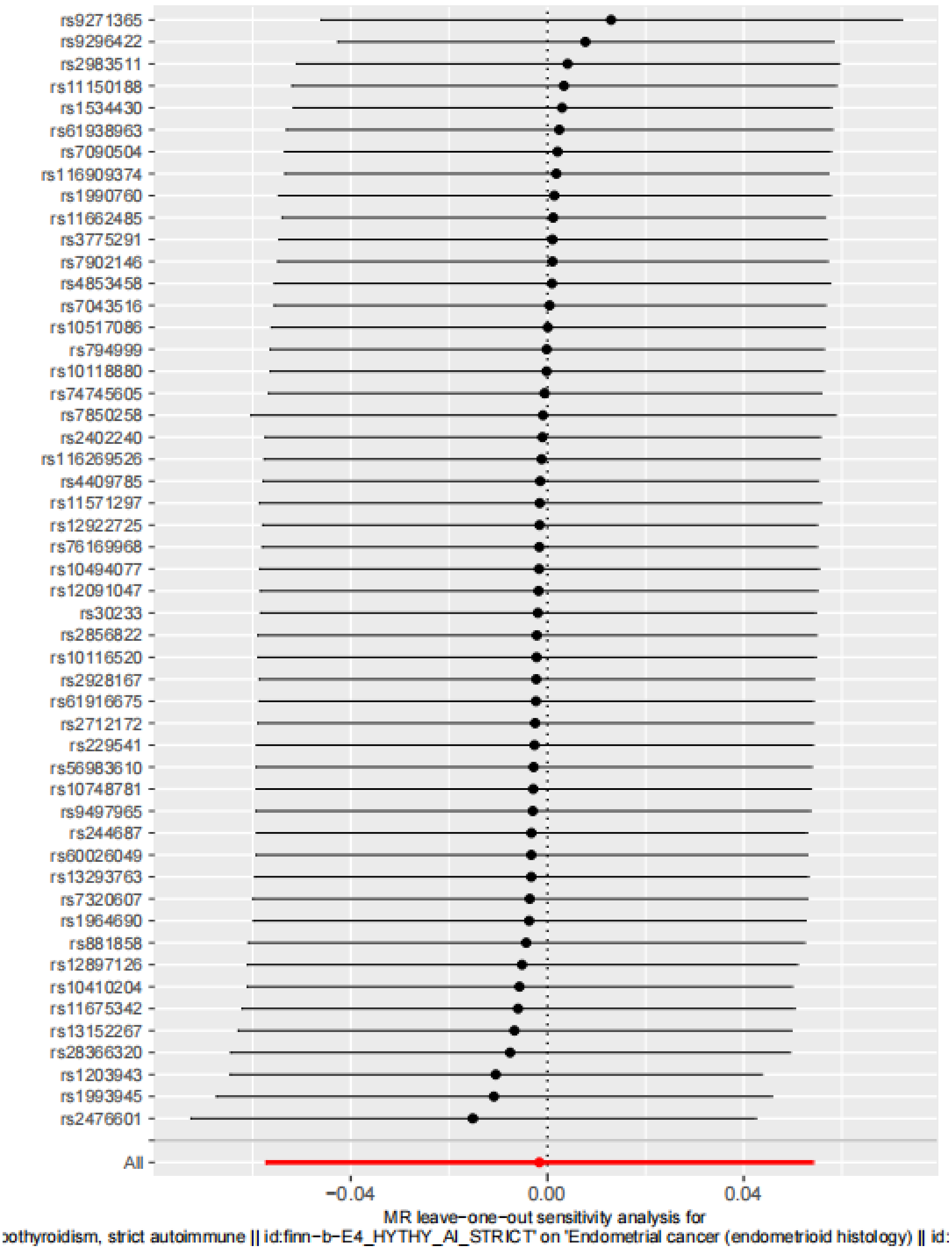

**Figure S8** Leave-one-out sensitivity analysis of the impact of autoimmune hypothyroidism on non-endometrioid endometrial cancer risk.

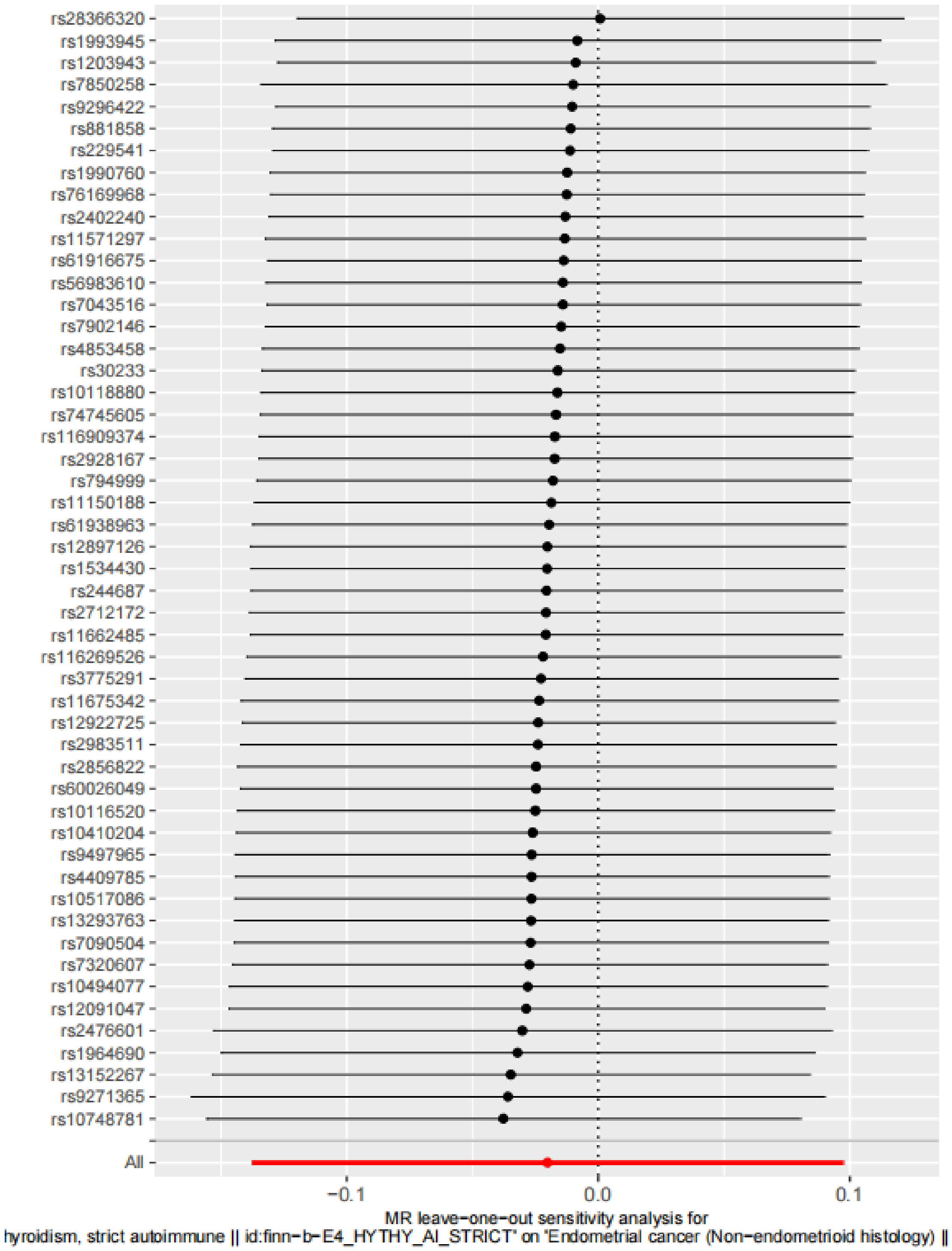

**Figure S9** Leave-one-out sensitivity analysis of the impact of endometrioid endometrial cancer on hypothyroidism.

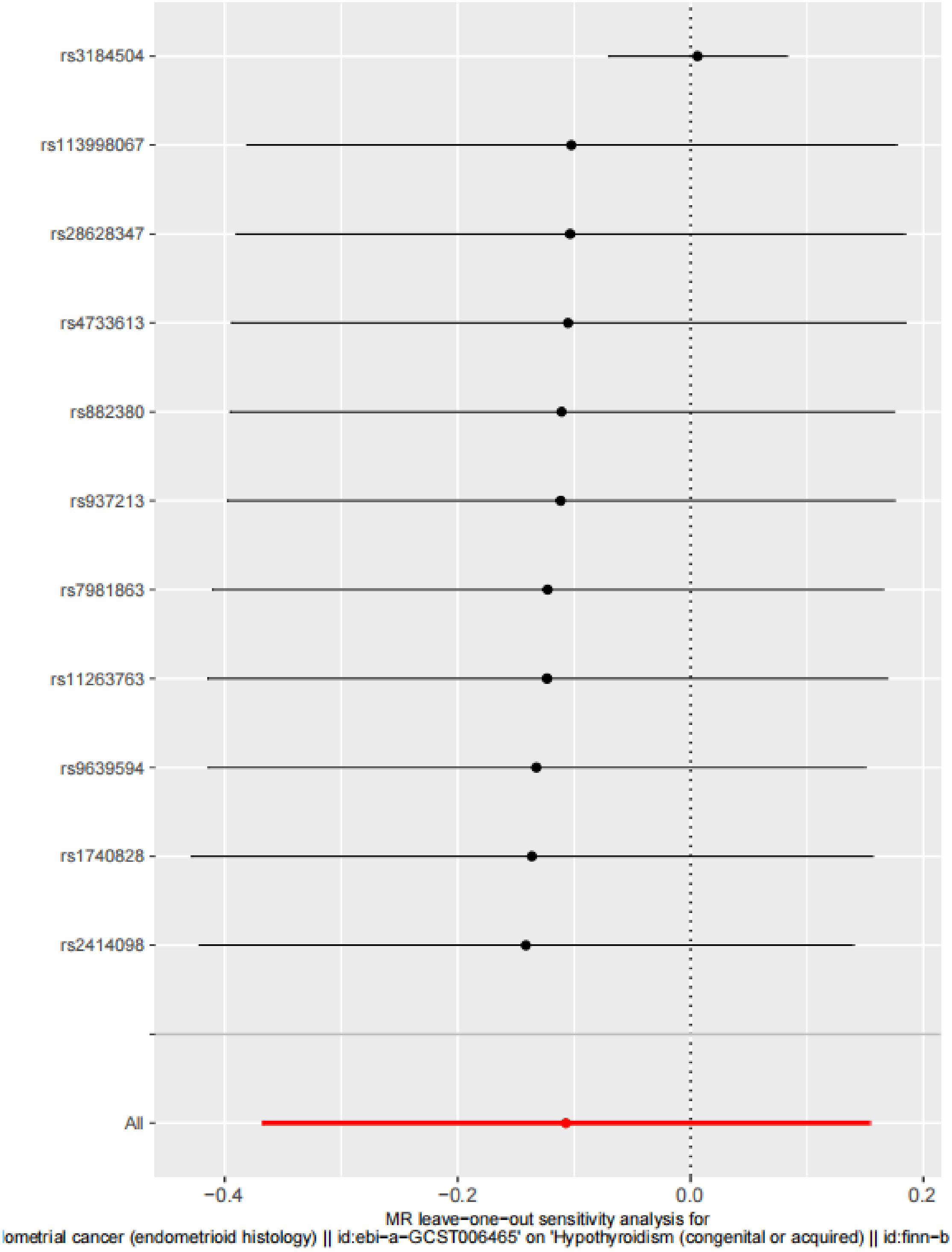

**Figure S10** Leave-one-out sensitivity analysis of the impact of endometrioid endometrial cancer on autoimmune hypothyroidism.

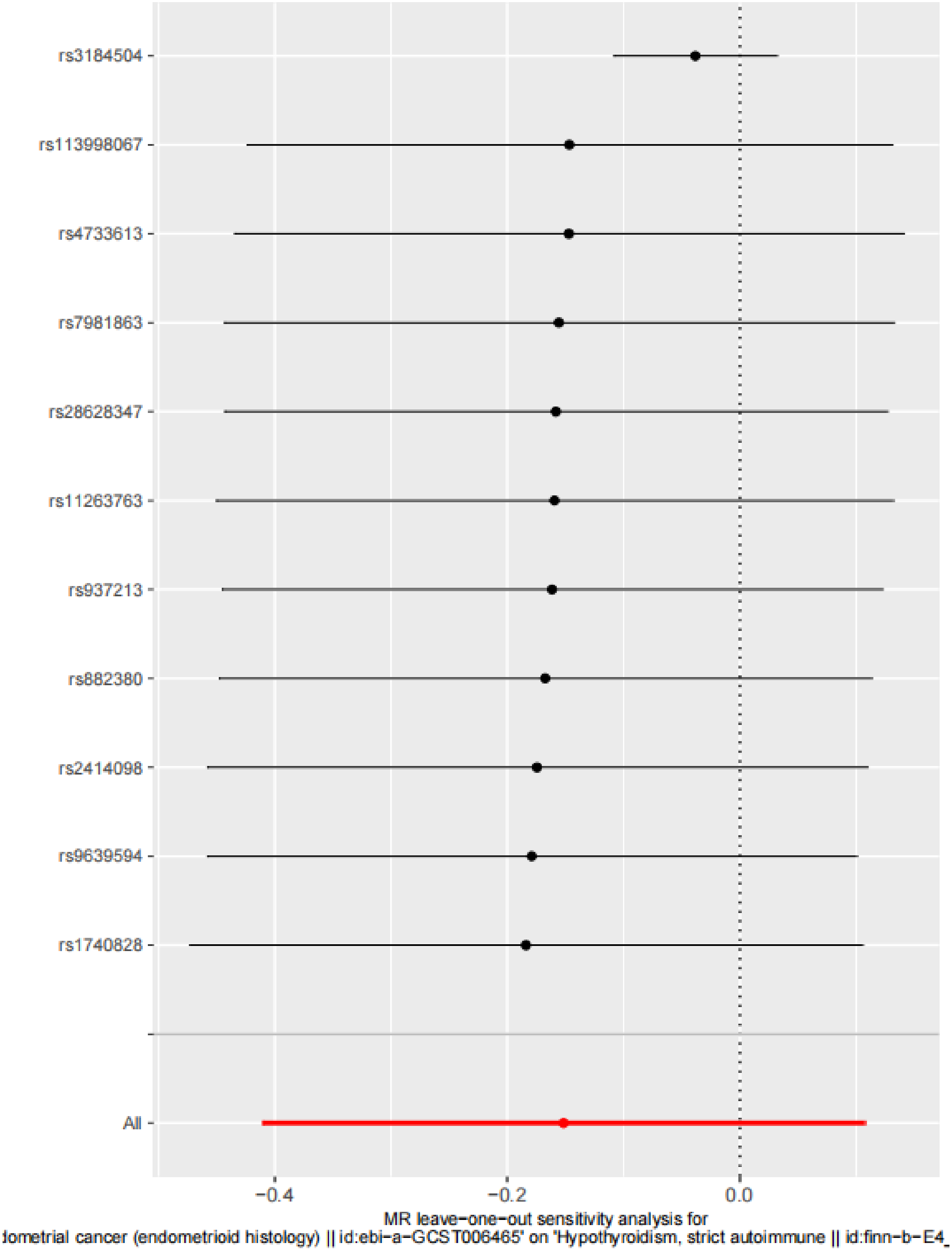

**Figure S11** Leave-one-out sensitivity analysis of the impact of non-endometrioid endometrial cancer on hypothyroidism.

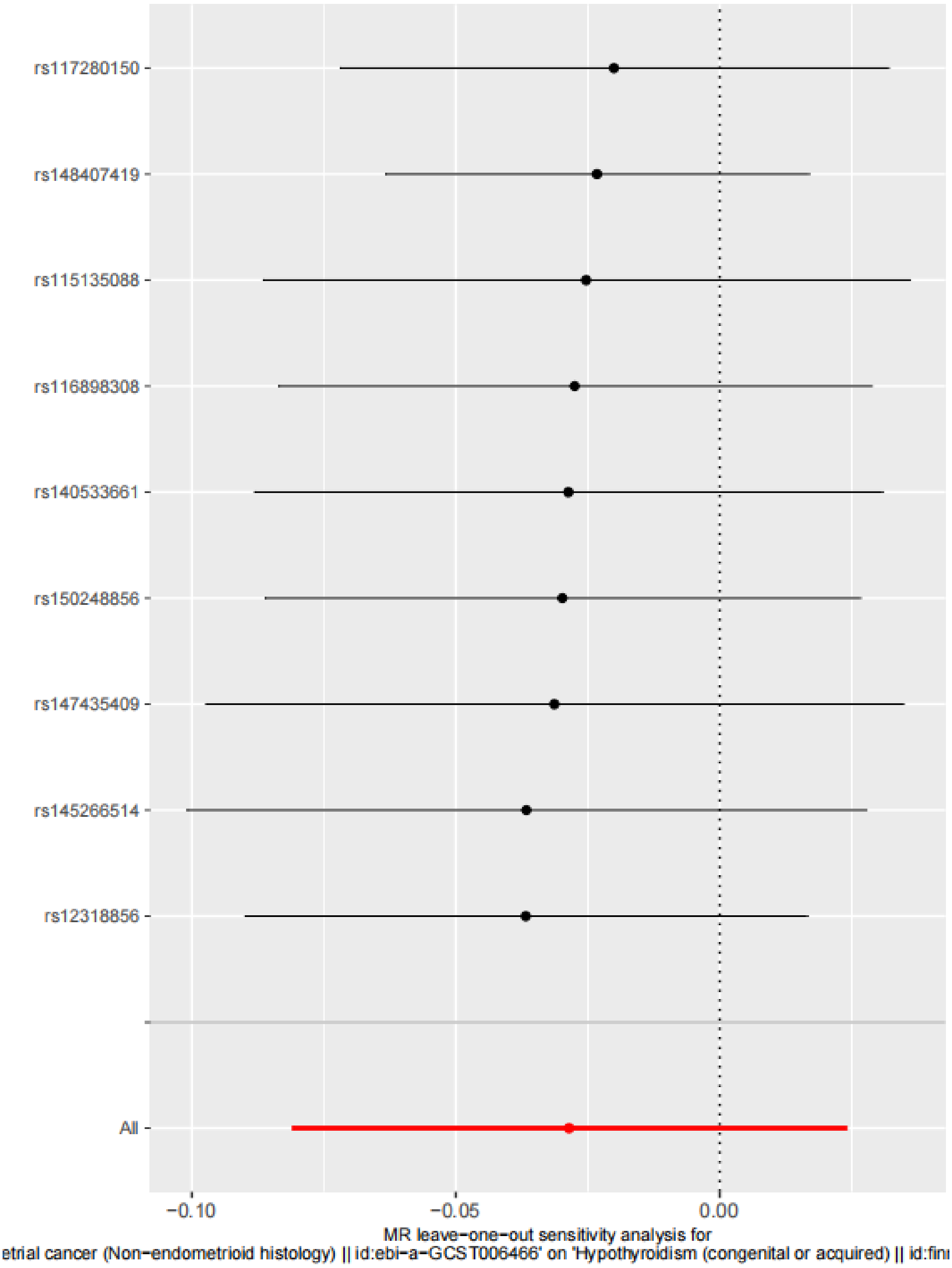

**Figure S12** Leave-one-out sensitivity analysis of the impact of non-endometrioid endometrial cancer on autoimmune hypothyroidism.

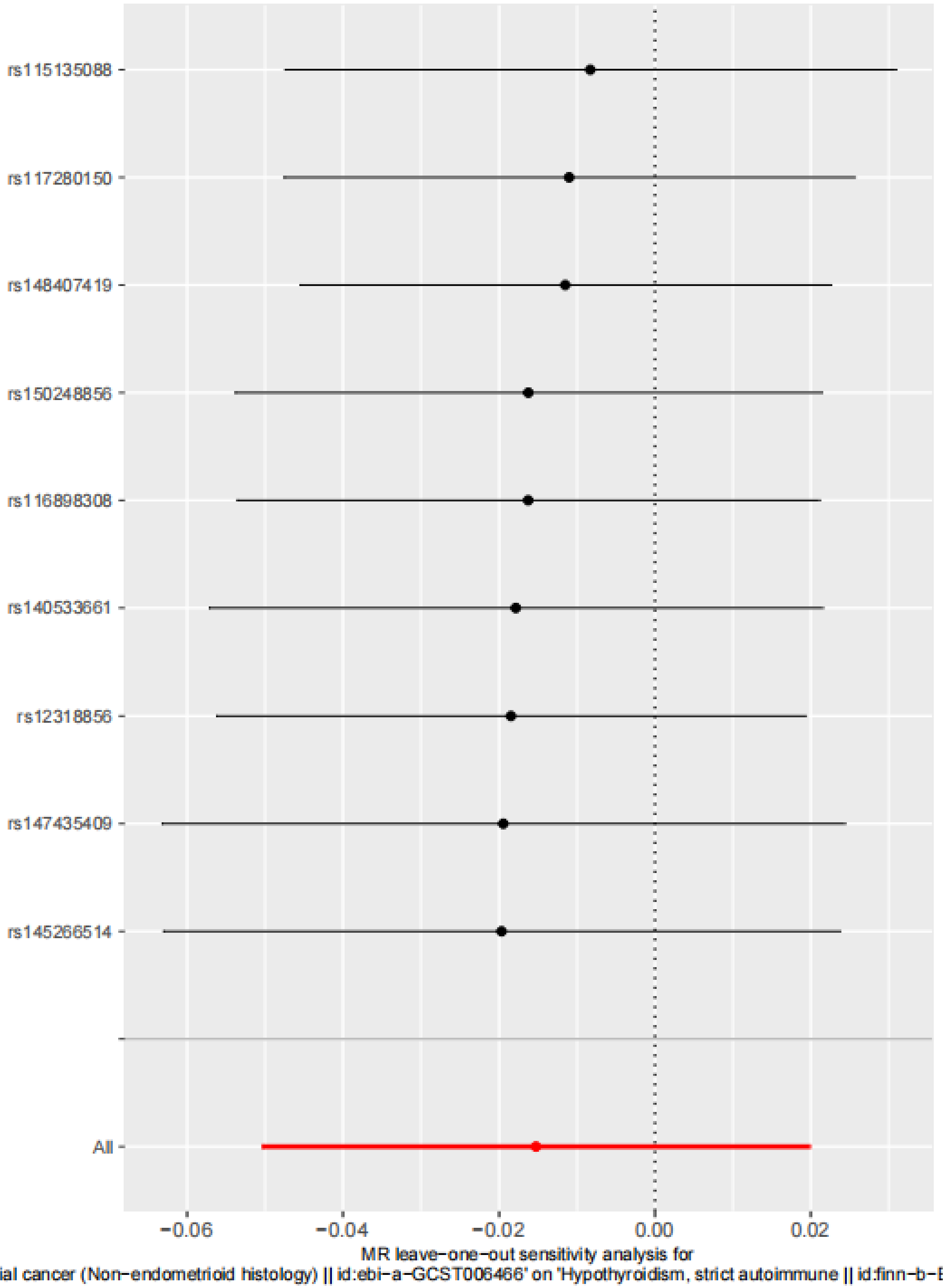

Supplement: Supplementary file 1 [file Image_1.pdf]
